# Long-Term Data From Patients Who Received Pembrolizumab in Locally Advanced or Metastatic Cutaneous Squamous Cell Carcinoma

**DOI:** 10.1155/crom/7038584

**Published:** 2025-02-23

**Authors:** Katrine Elsner Melgaard, Camilla Kjaer Lonkvist, Anni Linnet Nielsen, Dorte Lisbet Nielsen, Rikke Løvendahl Eefsen

**Affiliations:** Department of Oncology, Herlev Gentofte Hospital, Copenhagen, Denmark

**Keywords:** case report, checkpoint inhibitor, cutaneous squamous cell carcinoma, immunotherapy, pembrolizumab, programmed death-ligand 1 inhibitor (PD-L1 inhibitor)

## Abstract

Nonmelanoma skin cancer (NMSC) is one of the most common cancers worldwide and cutaneous squamous cell carcinoma (CSCC) is the second most prevalent type of the NMSCs. Most often, the prognosis is good when treated with surgery with or without additional radiotherapy; however, in about 1% of patients, the disease is inoperable or advanced with no curative potential. We present four cases in which the immune checkpoint inhibitor pembrolizumab was given to patients with advanced CSCC. Three patients obtained complete response (CR) with an ongoing duration of response with a follow-up time of 60, 78, and 79 months and one who achieved stable disease (SD) as the best response. Two of the patients discontinued treatment after eight cycles of pembrolizumab due to side effects. Immunotherapy with a programmed death protein 1 (PD-1) inhibitor is an approved therapy for these patients today. The successful treatment with long-term duration of response of these three out of four patients supports the use of a PD-1 inhibitor as a primary treatment for locally advanced and metastatic CSCC.

## 1. Introduction

Cutaneous squamous cell carcinoma (CSCC) is widely considered the second most common skin cancer in the world [1], affecting 700,000 people every year in the USA [2]. Although accurate incidence data are limited due to inadequate registration, the incidence seems to be increasing [3, 4]. The prognosis is often favorable for local CSCC treated with either surgery or radiotherapy with cure rates above 90% [5]. Patients with locally advanced or metastatic CSCC are often complicated to treat due to localization of the primary tumor in the head and neck area in 80% of the cases, several comorbidities, fragility, and high age [6]. These circumstances may challenge the use of chemotherapy as it may be poorly tolerated and the prognosis is often poor [7, 8]. Novel targeted agents have recently become a focus in the treatment of advanced CSCC, especially the epidermal growth factor receptor (EGFR) inhibitor cetuximab, as well as immune checkpoint inhibitors (ICIs) [1, 9]. Advances in molecular cancer biology have provided evidence that the immune system plays a pivotal role in the development and treatment of CSCC, as it has been observed that about 10% of high-risk CSCC express programmed death-ligand 1 (PD-L1) inhibitor and often have mutations [5, 8, 10].

It has been shown that the ICI, such as the programmed death protein 1 (PD-1) inhibitors, have a positive effect in CSCC, which led to the approval of cemiplimab, a PD-1 inhibitor, for patients with CSCC in Denmark April 2020 [11]. An FDA approval was achieved for the use of pembrolizumab in June 2020 for metastatic or recurrent CSCC based on data from the KEYNOTE-629 trial [12] and in July 2021 for locally advanced CSCC [13]. The safety profile of PD-1 inhibitors is well known with only mild to moderate side effects and thus presenting a more tolerable treatment in comparison to earlier biological candidates [1, 13, 14]. Complete response (CR) and partial response (PR) have been reported for several patients with CSCC, who received immunotherapy with cemiplimab [15, 16]. In the study by Rischin et al., a CR rate of 20.3% was reported for patients with metastatic CSCC. The median time to CR was 11 months, and for patients with CR or PR, an ongoing response was present at 12 months for 87.8% of the patients.

Here, we present long-term results of four cases of PD-1 blockade using intravenous pembrolizumab in patients with locally advanced or metastatic CSCC, who initiated treatment at our institution between December 2017 and October 2018. Patients were selected for immunotherapy based on a positive tumor proportion score (TPS) of PD-L1, which was the case for four patients in this period. The patients consented that their data could be used in this descriptive report. The treatment with pembrolizumab was assessed using fluorodeoxyglucose positron emission tomography-computerized tomography (FDG-PET/CT) and the Response Evaluation Criteria in Solid Tumors (RECIST) 1.1.

## 2. Case Presentation

### 2.1. Case No. 1

A 76-year-old man presented with recurrent CSCC in the lymph nodes, liver, lung, and bone metastases. Prior to pembrolizumab, he underwent surgery and radiation therapy. At recurrence, he received palliative chemotherapy with paclitaxel and capecitabine. After 11 cycles, he had progressive disease. The PD-L1 expression of tumor cells, assessed by the TPS, was 1%–50%. Due to PD-L1 positivity of the tumor cells, treatment with pembrolizumab was initiated. He received 11 cycles. Initially, regression of the lung metastases was observed on FDG-PET/CT scan, but the best overall response was stable disease. Side effects were Common Terminology Criteria for Adverse Events (CTCAE) Grade 1 diarrhea, Grade 2 adrenal insufficiency, and Grade 3 pneumonitis. The adrenal insufficiency and pneumonitis were treated with glucocorticoids, and treatment with pembrolizumab was ceased. Palliative radiotherapy was given to a lung metastasis, initially with stable disease for 6 months; however, 5 months after ceased pembrolizumab treatment, a control scan showed progressive disease with lung metastases as well as pneumonitis. Most likely, pneumonitis was a side effect of the prior pembrolizumab treatment, but radiotherapy may have contributed. The patient passed away due to disease progression, 1 year and 3 months after completion of the pembrolizumab treatment (see Table 1 for an overview of the patients).

### 2.2. Case No. 2

A 76-year-old man presented with a cutaneous basaloid squamous cell carcinoma near the orbit and a tumor growing close to the base of the skull. He underwent surgery and radiotherapy, and already 1 month after radiotherapy was completed, he had an inoperable relapse and received first-line paclitaxel and capecitabine. After 5 cycles, he had severe neurotoxicity, and the chemotherapy was discontinued. The tumor expressed PD-L1, with a TPS of 100%. At progression, he received pembrolizumab. After 8 cycles, pembrolizumab was discontinued due to Grade 2 colitis and arthritis. The side effects were well treated with glucocorticoids. FDG-PET/CT revealed he had a CR. To date, the duration of response (DOR) has been 78 months.

### 2.3. Case No. 3

A 79-year-old man presented with locally advanced CSCC in the parotid region with involvement of the mandibular bone, ear, and skin. Initial treatment was surgery and radiotherapy. Seven months after radiotherapy was completed, the patient had an inoperable relapse in the parotid region. The PD-L1 TPS was 20%. Due to PD-L1 positive tumor cells, pembrolizumab treatment was initiated. The patient received 36 cycles of pembrolizumab. A CR was achieved, evaluated on an FDG-PET/CT. The patient still has CR, with a follow-up time of 60 months. The only side effect was a Grade 1 pruritus.

### 2.4. Case No. 4

A 75-year-old man presented with CSCC on his clavicle and with metastases to the contralateral lymph nodes of the neck, left axillary fold, and direct growth into the sternocleidomastoid muscle. He underwent surgery. At relapse, he received 6 cycles of palliative chemotherapy with paclitaxel and capecitabine. Six months after, there was a local relapse and surgery was performed. Four months after surgery, progression of tumor was observed in the enlarged lymph nodes of the left neck. The PD-L1 expression based on the TPS was 100%. Due to the positive PD-L1 expression of tumor cells, pembrolizumab was initiated. He received 8 cycles, then the treatment was terminated due to Grade 3 aortitis, which was successfully treated with systemic corticosteroids. Additional side effects were Grade 1 pruritus and Grade 1 exanthema. After 8 cycles of pembrolizumab, an FDG-PET/CT revealed that the lymph nodes had disappeared. He achieved CR and still has CR, with a follow-up time of 79 months.

## 3. Discussion

The four patients with CSCC treated with pembrolizumab achieved either CR (*n* = 3) or stable disease (SD) (*n* = 1) as the best objective response according to RECIST 1.1. Three patients still have a CR whilst one patient passed away due to disease progression. ICI is an approved therapy for locally advanced, recurrent, and metastatic CSCC. The first data to support this was described by Migden et al., who observed an objective response rate (ORR) of 44% in patients with locally advanced CSCC without distant metastases, treated with cemiplimab [11]. The median DOR was not reached at the data cutoff, and the longest DOR was 24.2 months [11]. In the Keynote-629 trial, the ORR was 34.3% for patients receiving pembrolizumab, a CR was observed in 4 out of 130 patients, and a PR was observed in 32 patients [13]. In the long-term data for the Keynote-629 trial, ORR was 35.2% and CR was observed in 12.4% of the patients with recurrent or metastatic CSCC [17]. Median progression-free survival (PFS) was 14.4 months in the local advanced cohort and 5.7 months in the cohort with recurrent or metastatic disease, while the median DOR was 47.2 months in the locally advanced group and not reached in the recurrent and metastatic group. In phase 2 CARSKIN trial (NCT02883556), patients with locally advanced CSCC or metastatic CSCC, who had not received chemotherapy or anti-EGFR therapy, were enrolled. The median follow-up time was 22.4 months, and at the data cutoff, the ORR was 42%, including 4 out of 57 patients with CR and 20 patients with a PR. The PFS was 6.7 months, and the DOR was not reached [14]. These data are in line with the study by Rischin, as ORR was 50.8% for metastatic CSCC and a median DOR that had not been reached at 12 months for patients who had a CR or PR [16]. At the data cutoff for our case series on 30 December 2024, the follow-up time was 60, 78, and 79 months for the three patients with CR and DOR was not reached. All patients still have a CR, which is in line with the CARSKIN trial. The observation that three out of four patients still have a CR is noteworthy and in line with the long-term data presented in trials and in real-world populations [16, 18]. Of note, it has been observed that treatment with cemiplimab gave a PR in almost all patients treated and a median time to CR of 11 months [16]. In the real-world population from Australia and New Zealand, including a total of 286 patients with locally advanced or metastatic CSCC, an ORR of 60% was observed for patients who received either cemiplimab or pembrolizumab [18]. Also, in a single-center retrospective study, in elderly patients receiving cemiplimab or pembrolizumab, the ORR was 57% [19]. Furthermore, in an Italian retrospective cohort of elderly patients, with a median age of 81 years, the ORR was 76.7% [20]. These data are in line with the findings of the patients we treated at our institution as patients were old and with some comorbidities. The patients in our case series were not immunocompromised, which was the case in one of the cohorts of elderly patients with CSCC treated with ICI [15]. In a meta-analysis of antineoplastic therapies, cemiplimab was compared to other systemic therapies for advanced CSCC, and a PFS of 18.4 months was observed in patients who received cemiplimab in comparison to other systemic therapies. For pembrolizumab, the estimated median PFS was 14.2 months using this model [21].

The durable responses reported in clinical trials [16, 22], and observed in the patients included in this long-term analysis, may be useful in the future management of CSCC patients, as many patients have a localized tumor in the head and neck region, which can be difficult to handle with surgery due to morbidity and disfigurement. As reviewed in the study by McLean et al. [22], 7 of the 119 patients declined to have surgery involving the orbital region. The patients received therapy with pembrolizumab or cemiplimab. Six of the patients had response to immunotherapy and avoided orbital surgery. The patients in our case series had their primary tumor located in the head and neck region, and all patients had surgery and radiation therapy performed at the time of diagnosis.

Treatment with pembrolizumab may give autoimmune toxicities [23, 24]. Three patients in this case report experienced side effects to pembrolizumab, one patient had aortitis CTCAE Grade 2, one patient had colitis and arthritis CTCAE Grade 2, and one patient had adrenal insufficiency CTCAE Grade 2 as well as pneumonitis CTCAE Grade 3. For all three patients, pembrolizumab treatment was ended and therapy with glucocorticoids was initiated. The toxicities observed when patients were treated with pembrolizumab were manageable when treated with glucocorticoids.

In the ongoing DESQUAMATE trial, NCT05025813, pembrolizumab is given as neoadjuvant therapy, and it will be interesting if patients can avoid surgery after ICI [25]. In the KEYNOTE-630 trial, NCT03833167, pembrolizumab was given after surgery and radiotherapy as adjuvant therapy in patients with high-risk locally advanced CSCC. The trial was discontinued in 2024, due to negative results [26]. The C-POST trial (NCT0396004) is an ongoing phase 3 trial, to evaluate cemiplimab as adjuvant therapy in CSCC patients. ICI is thus investigated in multiple settings, neoadjuvant, adjuvant, and in metastatic setting, and it will be interesting to see the results. In conclusion, this case report shows long-term durable responses, with a promising effect with the use of pembrolizumab as the treatment of advanced CSCC.

## Figures and Tables

**Table 1:** Overview of patients and response to treatment.

	**Case 1**	**Case 2**	**Case 3**	**Case 4**	**Overall**
Gender	M	M	M	M	M (*n* = 4)
Age (years)	76	76	79	75	Range: 75–79
Stage of disease	IV	III	III	III	III–IV
Number of cycles with pembrolizumab	11	8	24	8	Range: 8–24
Location of primary tumor	Right parotid region	Left orbitae	Right parotid region	Right axillae	NA
Best objective response (RECIST 1.1)	SD	CR	CR	CR	CR (*n* = 3), SD (*n* = 1)
Pembrolizumab treatment line	2	2	2	2	2 (*n* = 4)
Prior surgery	Yes	Yes	Yes	Yes	Yes (*n* = 4)
Prior radiation therapy	Yes	Yes	Yes	No	Yes (*n* = 3), No (*n* = 1)
Treatment related adverse events (TRAE), Grade 1	Grade 1: diarrhea	None	Grade 1: pruritus	Grade 1: pruritus and rash	Grade 1 (*n* = 3)
TRAE, Grade 2	Grade 2: adrenal insufficiency	Grade 2: colitis and arthritis	None	Grade 2: aortitis	Grade 2 (*n* = 3)
TRAE, Grade 3	Grade 3: pneumonitis	None	None	None	Grade 3 (*n* = 1)
Time to progression	5 months	No progression	No progression	No progression	PD (*n* = 1), No PD (*n* = 3)
Duration of response	5 months	Not reached	Not reached	Not reached	5–not reached
Follow-up time	5 months	78 months	60 months	79 months	5–79 months
Immunocompromised (yes/no)	No	No	No	No	No
Charlson comorbidity index	10	6	6	10	8 (6–10)

Abbreviation: NA: not applicable.

## Data Availability

The data that support the findings of this study are available from the corresponding author upon reasonable request.

## Nomenclature

CSCC: cutaneous squamous cell carcinoma
CR: complete response
CT: computed tomography
DOR: duration of response
EGFR: epidermal growth factor receptor
NMSC: nonmelanoma skin cancer
CTCAE: Common Terminology Criteria Adverse Events
ORR: objective response rate
PD-L1: programmed death-ligand 1
PD-1: programmed death protein 1
PET/CT: positron emission tomography/computed tomography
PFS: progression-free survival
PR: partial response
RECIST 1.1: Response Evaluation Criteria in Solid Tumors 1.1
